# Ischemic Stroke as the Initial Presentation of Advanced Pancreatic Adenocarcinoma: A Case of Marantic Endocarditis

**DOI:** 10.7759/cureus.103844

**Published:** 2026-02-18

**Authors:** Micah Harris

**Affiliations:** 1 Internal Medicine, OhioHealth Riverside Methodist Hospital, Columbus, USA

**Keywords:** embolic stroke of undetermined source, hypercoagulability state, marantic endocarditis, metastatic pancreatic adenocarcinoma, non-bacterial thrombotic endocarditis

## Abstract

Nonbacterial thrombotic endocarditis (NBTE), formerly known as marantic endocarditis, is a rare condition characterized by sterile vegetations on cardiac valves and is often associated with hypercoagulable states such as adenocarcinoma. We describe a patient who presented with multiple embolic cerebrovascular accidents, ultimately found to be secondary to NBTE, which served as the initial manifestation of previously undiagnosed advanced pancreatic adenocarcinoma. This case underscores the importance of considering NBTE in patients with multifocal embolic infarcts, particularly when blood cultures are negative, echocardiography reveals valvular abnormalities without evidence of infection, and clinical suspicion for an underlying malignancy is present.

## Introduction

Nonbacterial thrombotic endocarditis (NBTE), formerly known as marantic endocarditis, is a rare formation of vegetations on the cardiac valves associated with states of hypercoagulability such as malignancy and inflammatory disorders [1,2]. Although the exact pathogenesis of NBTE remains unknown, it is suspected to be a process of endothelial dysfunction caused by oxidative stress, inflammatory cytokines, and shear stress. The resulting endothelial damage leads to the formation of fibrin-platelet-rich thrombi on the cardiac valve, exaggerated by states of hypercoagulability [1]. Among the malignancies most frequently associated with NBTE are the mucin-producing adenocarcinomas of the lung, ovary, biliary system, pancreas, breast, and stomach [3]. Initial presentation of NBTE can vary between cerebrovascular accident (CVA), shortness of breath, chest pain, and acute limb ischemia, although CVA often accounts for around half of presentations [1]. In this case, we describe how the first presentation of advanced pancreatic adenocarcinoma was an embolic CVA from NBTE. It showcases how the hypercoagulability of malignancy can initially present as a stroke and the importance of recognizing evidence of embolic showering on imaging to prompt early evaluation with echocardiography and malignancy screening.

## Case presentation

**Figure 1:**
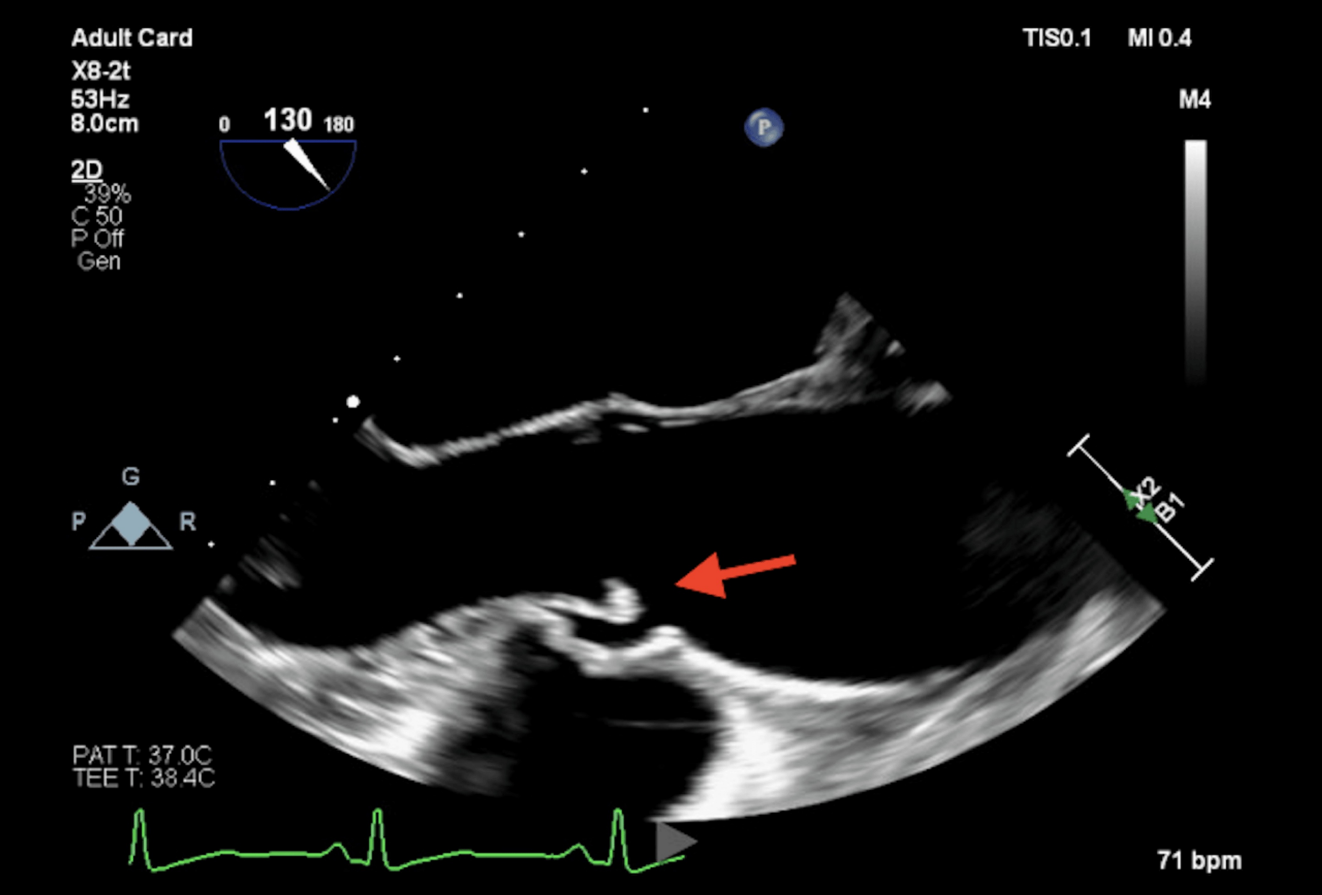
Nonbacterial thrombotic endocarditis on transesophageal echocardiography. Transesophageal echocardiography findings in the midesophageal aortic valve long axis view with mild thickening and prominent focal thickening of the tip of the right coronary cusp (red arrow) were suggestive of nonbacterial thrombotic endocarditis.

A 56-year-old female with a past medical history of tobacco use presented to the emergency department (ED) with six days of progressive expressive aphasia. She had no history of atrial fibrillation, prior transient ischemic attack, hypertension, or illicit drug use. Initial computed tomography (CT) and computed tomography angiography (CTA) of the head were unremarkable. Magnetic resonance imaging (MRI) of the head demonstrated multiple bilateral foci of restricted diffusion involving multiple vascular territories consistent with shower emboli. She received an aspirin load and was transferred to a tertiary center. Neurology initiated aspirin and statin therapy, although hemoglobin A1c and lipid panel were unremarkable. Transthoracic echocardiogram (TTE) revealed an ejection fraction of 63%, no intracardiac shunt, mild aortic regurgitation, and no clear cardiac source of emboli. Her speech improved during admission, and although a transesophageal echocardiogram (TEE) was offered, she declined and was discharged with plans for outpatient stroke clinic follow-up. A few weeks later, at her follow-up stroke visit, she was found to have new deficits, including new deconjugate gaze, left gaze nystagmus, and diplopia, prompting return to the ED and activation of a level 1 stroke alert. She was loaded with clopidogrel. Repeat CT and CTA remained unremarkable, while MRI again demonstrated scattered acute infarcts. A TEE was performed, which showed mild aortic valve thickening with focal thickening at the tip of the right coronary cusp (Figure 1). Cardiology noted the appearance to be atypical for infectious endocarditis, and blood cultures were obtained the same day, which remained negative after full incubation. Antibiotic therapy was deferred, given the patient's lack of infectious symptoms, normal vital signs, normal white blood cell count, and TEE findings suggestive of NBTE.

**Figure 2:**
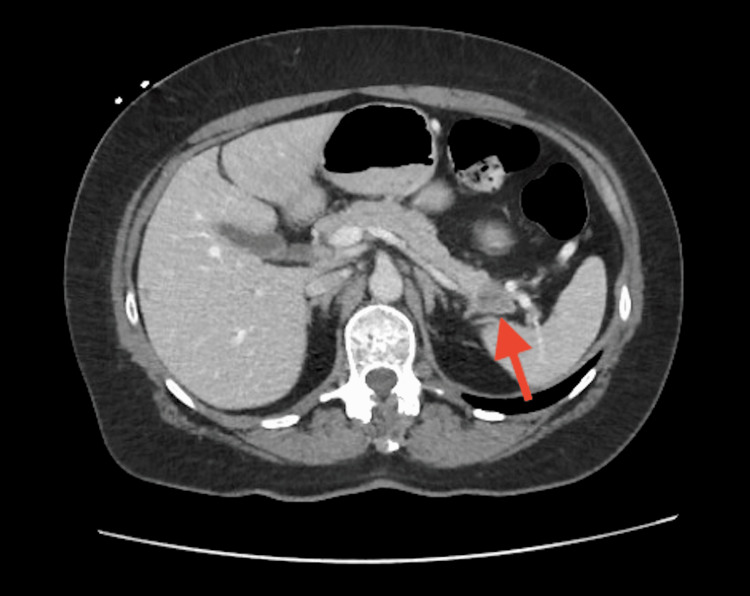
Pancreatic malignancy on CT of the abdomen with contrast. Malignancy screening with CT of the chest, abdomen, and pelvis was significant for a distal pancreatic body/tail mass (red arrow) concerning for a primary malignancy and multiple liver lesions concerning for metastasis.

The patient was started on dabigatran, and a hypercoagulability panel, including cardiolipin antibodies, lupus anticoagulant, and IgG/IgM beta-2 glycoprotein antibodies, was unremarkable. CT of the abdomen/pelvis with contrast revealed a mass in the distal pancreatic body/tail and multiple low-density liver lesions concerning for metastatic malignancy (Figure 2). Tumor markers were markedly elevated, and endoscopy with fine needle aspiration confirmed adenocarcinoma.

Oncology was consulted, and implantable central venous access was placed with plans for beginning outpatient palliative systemic chemotherapy. She was discharged on dabigatran for anticoagulation after cost prohibited other anticoagulation options. Only a few days after discharge, the patient presented to the hospital again with new deficits secondary to a left middle cerebral artery occlusion. The patient's home dabigatran was held, and she underwent manual aspiration thrombectomy. Her post-procedural course was complicated by a large intraparenchymal hemorrhage, subarachnoid hemorrhage, and acute ischemia of the right lower extremity. Given her poor neurological status and complications, the family elected to transition to comfort-focused care.

## Discussion

In summary, our case illustrated NBTE presenting as multiple CVAs in the setting of unknown advanced pancreatic adenocarcinoma. Although a rare diagnosis, there have been reviews to elucidate the epidemiology of NBTE. In a large systematic review, NBTE was reported in 450 patients with a median age of 48, with a female to male ratio of 2:1. Embolic events occurred in about 70% of cases and presented most commonly as stroke and cancer, as the etiology was associated with higher mortality [4]. As hypercoagulability is thought to play a role in the formation of NBTE, it follows that one could expect pancreatic cancer to have the ability to present as NBTE, given the well-documented association of hypercoagulability with adenocarcinomas and secreted inflammatory cytokines [5]. Although this hypercoagulability is often seen with pancreatic cancer, NBTE is rarely reported antemortem. Rather, pancreatic cancer typically presents as asthenia, anorexia, weight loss, abdominal pain, and choluria/jaundice-related symptoms [6,7]. This case is an example of a rare presentation of advanced pancreatic cancer, as our patient did not display most of the typical expected symptoms. This case reminds the importance of recognizing a possible cause of showering emboli on imaging, as most patients with CVA in the setting of NBTE will have MRI evidence of multifocal bi-hemispheric ischemic lesions involving both the anterior and posterior circulations, as was seen in our case [8]. Antemortem diagnosis of NBTE remains a challenge because the vegetations are often small, friable, may embolize before visualization, and can produce no or little valvular dysfunction or murmurs. Accordingly, TEE is usually necessary as TTE is often undiagnostic, as was seen in our patient, who first underwent TTE that was undiagnostic [9]. Also, aortic valve thickening has a broad differential diagnosis that includes degenerative calcific aortic valve disease, infective endocarditis, NBTE, autoimmune-mediated valvulitis such as Libman-Sacks endocarditis, rheumatic heart disease, radiation-associated valvulopathy, and, less commonly, primary valvular tumors [10]. Further adding to the difficulty of diagnosis is the lack of universally accepted formal diagnostic criteria for NBTE. Nevertheless, most experts recommend a diagnostic framework combining a predisposing hypercoagulable condition, clinical evidence of systemic embolization, visualization of valvular vegetations, and exclusion of infective endocarditis through persistently negative blood cultures and absence of systemic infection [1,9]. In our case, the vegetation was discovered on TEE suggestive of NBTE, blood cultures remained negative, infective endocarditis was ruled out due to failure to meet the modified Duke criteria for diagnosis of infective endocarditis, and advanced pancreatic adenocarcinoma was discovered as a cause of hypercoagulability [11]. Historically, the mainstay of therapy for NBTE has been with unfractionated heparin or low molecular weight heparin (LMWH), usually with indefinite duration and treatment of the underlying cause. Warfarin is not preferred in malignancy-associated NBTE, given the poor control of hypercoagulability and recurrent emboli, and there is limited evidence for newer anticoagulants [12]. Surgical intervention is also not recommended unless the patient is in acute congestive failure [13]. Our patient was discharged on dabigatran, a direct thrombin inhibitor, chosen partly due to cost constraints, and was planning on palliative systemic chemotherapy of the pancreatic adenocarcinoma as the underlying cause for her presentation.

## Conclusions

NBTE should be considered in cryptogenic multifocal embolic stroke, prompting early echocardiography. Given its association with hypercoagulable adenocarcinomas and tumor-secreted cytokines, similar presentations warrant malignancy screening. Our case highlights that early TEE, anticoagulation, and cancer evaluation can expedite diagnosis in rapidly progressive, recurrent strokes. NBTE should remain on the differential in patients with embolic stroke and suspected or known malignancy.

## References

[1] Ahmed O, King NE, Qureshi MA (2025). Non-bacterial thrombotic endocarditis: a clinical and pathophysiological reappraisal. Eur Heart J.

[2] Al Chalaby S, Makhija RR, Sharma AN, Majid M, Aman E, Venugopal S, Amsterdam EA (2022). Nonbacterial thrombotic endocarditis: presentation, pathophysiology, diagnosis and management. Rev Cardiovasc Med.

[3] Itzhaki Ben Zadok O, Spectre G, Leader A (2022). Cancer-associated non-bacterial thrombotic endocarditis. Thromb Res.

[4] Alhuarrat MA, Garg V, Borkowski P (2024). Epidemiologic and clinical characteristics of marantic endocarditis: a systematic review and meta-analysis of 416 reports. Curr Probl Cardiol.

[5] Campello E, Ilich A, Simioni P, Key NS (2019). The relationship between pancreatic cancer and hypercoagulability: a comprehensive review on epidemiological and biological issues. Br J Cancer.

[6] Jameson GS, Ramanathan RK, Borad MJ, Downhour M, Korn R, Von Hoff D (2009). Marantic endocarditis associated with pancreatic cancer: a case series. Case Rep Gastroenterol.

[7] Porta M, Fabregat X, Malats N (2005). Exocrine pancreatic cancer: symptoms at presentation and their relation to tumour site and stage. Clin Transl Oncol.

[8] Oakley CI, Wysokinska EM, Kaminska A (2025). Topography of ischemic strokes in cancer-associated non-bacterial thrombotic endocarditis: a single-institution descriptive case series. J Stroke Cerebrovasc Dis.

[9] Tonutti A, Scarfò I, La Canna G, Selmi C, De Santis M (2023). Diagnostic work-up in patients with nonbacterial thrombotic endocarditis. J Clin Med.

[10] Otto CM, Nishimura RA, Bonow RO (2021). 2020 ACC/AHA guideline for the management of patients with valvular heart disease: a report of the American College of Cardiology/American Heart Association Joint Committee on clinical practice guidelines. J Am Coll Cardiol.

[11] Baddour LM, Wilson WR, Bayer AS (2015). Infective endocarditis in adults: diagnosis, antimicrobial therapy, and management of complications: a scientific statement for healthcare professionals from the American Heart Association. Circulation.

[12] el-Shami K, Griffiths E, Streiff M (2007). Nonbacterial thrombotic endocarditis in cancer patients: pathogenesis, diagnosis, and treatment. Oncologist.

[13] Asopa S, Patel A, Khan OA, Sharma R, Ohri SK (2007). Non-bacterial thrombotic endocarditis. Eur J Cardiothorac Surg.

